# *In Vivo* Ribbon Mobility and Turnover of Ribeye at Zebrafish Hair Cell Synapses

**DOI:** 10.1038/s41598-017-07940-z

**Published:** 2017-08-07

**Authors:** Cole W. Graydon, Uri Manor, Katie S. Kindt

**Affiliations:** 1 Synaptic Physiology Section, National Institute of Neurological Disorders and Stroke, National Institutes of Health, Bethesda, MD 20892 USA; 2 Section on Organelle Biology, Eunice Kennedy Shriver National Institute of Child Health and Human Development, National Institutes of Health, Bethesda, MD 20892 USA; 3 Section on Sensory Cell Development & Function, National Institute on Deafness and Other Communication Disorders, National Institutes of Health, Bethesda, MD 20892 USA; 4 Waitt Advanced Biophotonics Center, Salk Institute for Biological Studies, La Jolla, CA 92037 USA

## Abstract

Ribbons are presynaptic structures that mediate synaptic vesicle release in some sensory cells of the auditory and visual systems. Although composed predominately of the protein Ribeye, very little is known about the structural dynamics of ribbons. Here we describe the *in vivo* mobility and turnover of Ribeye at hair cell ribbon synapses by monitoring fluorescence recovery after photobleaching (FRAP) in transgenic zebrafish with GFP-tagged Ribeye. We show that Ribeye can exchange between halves of a ribbon within ~1 minute in a manner that is consistent with a simple diffusion mechanism. In contrast, exchange of Ribeye between other ribbons via the cell’s cytoplasm takes several hours.

## Introduction

Ribbon synapses are present in hair cells of auditory, vestibular and lateral line organs as well as in photoreceptors and bipolar cells of the visual system. These cells typically do not generate action potentials; rather, they translate graded changes in membrane potential into modulations of synaptic vesicle release at ribbon synapses onto postsynaptic neurons. The ultrastructural hallmark of these synapses is a specialized proteinaceous presynaptic structure, called a “ribbon”, which ranges in size (from tens to hundreds of nanometers) and shape (from spheres to flat plates)^1^. While many different proteins localize to the ribbon synapse^2, 3^, the ribbon itself is largely composed of the ribbon-specific protein Ribeye (~67% or more of the ribbon volume)^4–6^.

At the presynaptic active zone, ribbons tether and organize a pool of synaptic vesicles adjacent to clusters of L-type calcium channels. Based on the close association between ribbons and vesicles, the ribbon may serve as a synaptic vesicle “conveyor belt” or as a scaffold for compound fusion of vesicles (“safety belt”)^7, 8^, and may perform key steps in preparing synaptic vesicles for fusion (“priming”)^9, 10^. In addition to these proposed roles in organizing and preparing vesicles for fusion, evidence of correlations between ribbon ultrastructure and synaptic output properties has also mounted. For example, ribbon numbers and diameters vary tonotopically along auditory organs^11–13^, and ribbon size at each synapse within a hair cell correlates with spontaneous firing rates of postsynaptic auditory fibers^14, 15^.

Perhaps counterintuitive to the ordered and stereotypical patterns of ribbon number and size across auditory organs, electron microscopy evidence also suggests that individual ribbons are structurally dynamic, particularly during assembly/disassembly^16^ processes – as occurs during development^17–19^, hibernation^20^, and diurnal cycles^21–24^. While the discrete time points observed in electron micrographs often exhibit dramatic ribbon morphologies, suggestive of a certain degree of ultrastructural plasticity, the temporal resolution of these observations is relatively poor (e.g. minutes and longer). As a result, the real-time dynamics of the ribbon’s ultrastructure, and its interactions with tethered synaptic vesicles, remain largely unknown.

In this study, we investigate *in vivo* real-time structural dynamics at ribbons in zebrafish lateral line hair cells. To do this, we utilize a transgenic *ribeye b*-EGFP line^25^ that labels the ribbon’s main structural protein (Ribeye) with a fluorescent probe. Our findings suggest a relatively slow synaptic Ribeye turnover rate for whole ribbons (~6–7 hours), but an internal mobility of Ribeye within these large spherical ribbons on timescales of a few minutes.

## Results

**Figure 1:**
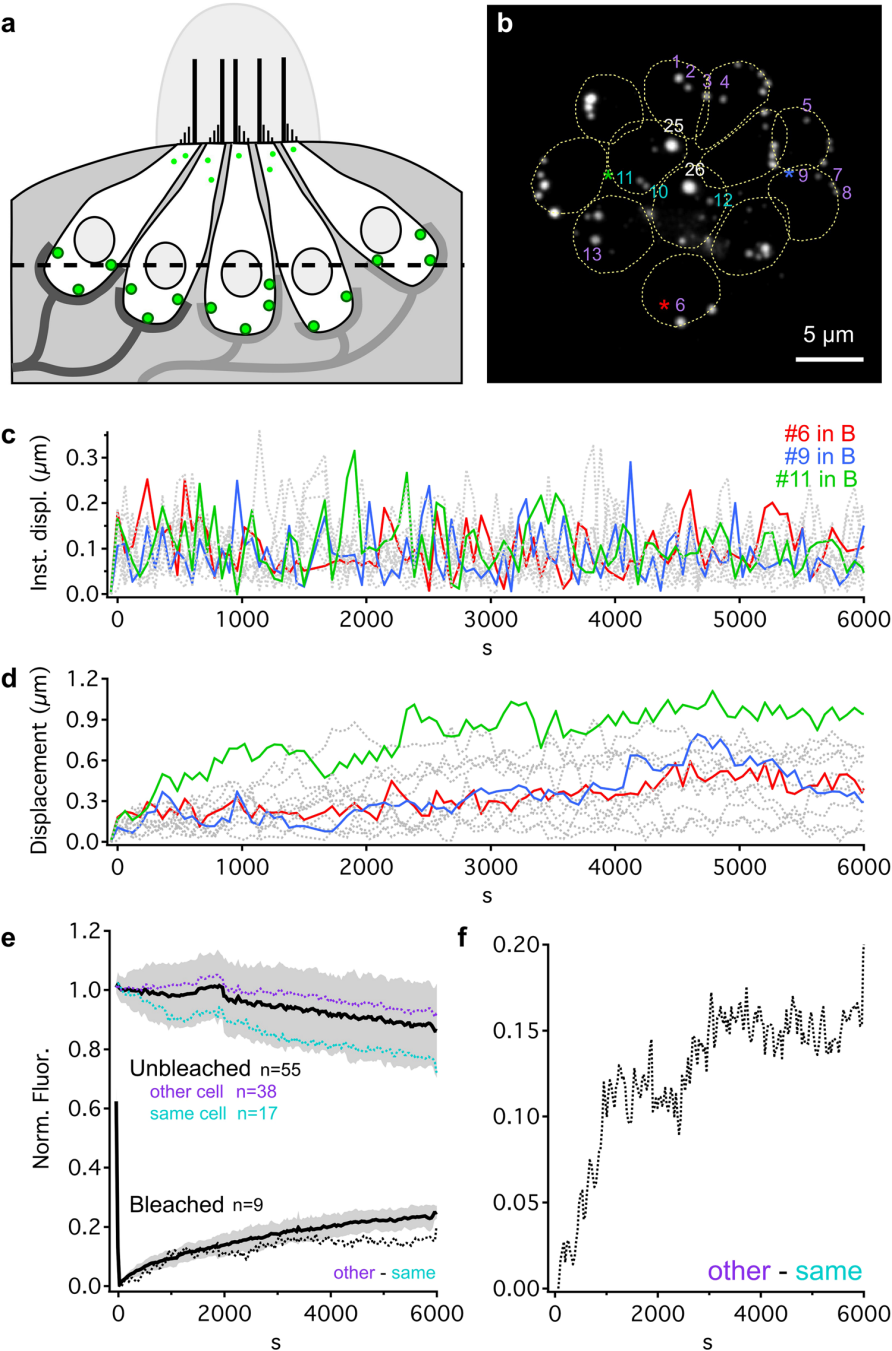
Long term tracking of ribbon movements, Ribeye turnover and exchange. (**a**) Schematic cross-section of a Ribeye b-EGFP neuromast, showing five hair cells contacted by nerve fibers at ribbon synapses (green). Some small Ribeye aggregates accumulate at the apical surface of the hair cells - these were excluded from analysis. The dotted line corresponds to the imaging plane shown in b. (**b**) Example maximum intensity projection of a whole Ribeye b-EGFP neuromast (top-down view). Numbers indicate specific ribbons, spread across the neuromast, used for analysis in **c–f**. Dotted: cell outlines. Colors correspond to **c–f**. Ribbons “25” and “26” were bleached (white). (**c)** Instantaneous displacements calculated for movements made between image acquisitions (~1 min interval) for the length of our experiments. Red, blue, and green traces correspond to ribbons (labeled with *) in **b**. Other labeled ribbons in b are dashed gray. (**d**) Same ribbons as c, showing accumulated X-Y displacements through time. (**e**) Normalized average fluorescence intensity through time following bleaching of whole ribbons (bold black ± SD in gray). Unbleached ribbons were sorted as either 1) in the same cell as a bleached ribbon (teal) or, 2) in a cell with no bleached ribbons (purple). Dashed black line: difference between “same cell” and “other cell” lines. (**f**) Zoomed view of difference between “same cell” and “other cell” lines from **e**. See also Supplementary Fig. S1.

**Figure 2:**
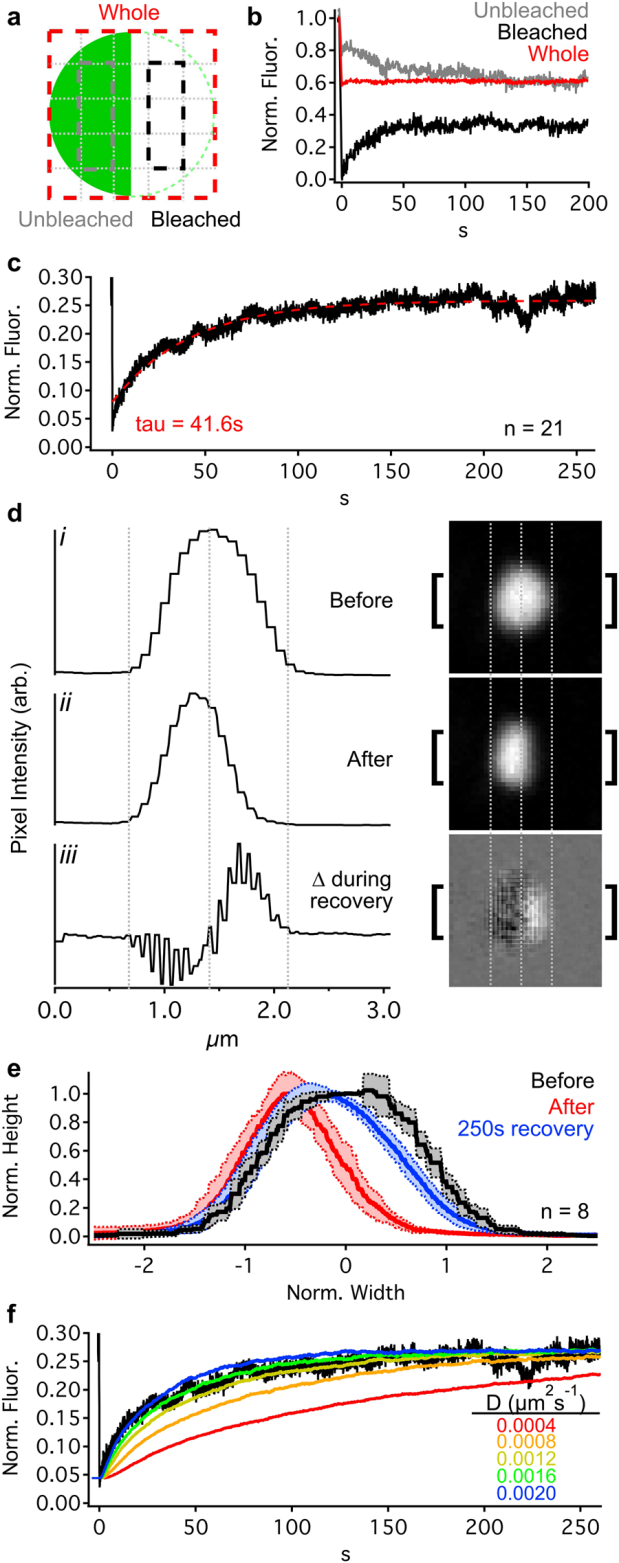
Ribeye is dynamic and mobile within the ribbon. (**a**) Ribbon schematic showing measurement regions: the whole ribbon (dashed red), within the bleached (dashed black) and unbleached (dashed gray) hemispheres. (**b**) Normalized fluorescence profiles for each region in a following bleaching of half the ribbon (at 0 s) for an example ribbon (same as in d). (**c**) Average normalized recovery curve for bleached hemisphere (n = 21 ribbons; red: exponential fit). (**d**) Pixel intensity profiles (left) from regions between brackets in images (right) before (i) and after (ii) bleaching. The bottom profile and image (iii) show the average change in pixel intensity for each pixel over successive images during the first 50 time points (~17 s) of recovery. Gray indicates no average change, while brighter and darker values indicate gain and loss of fluorescence, respectively. (**e**) Pixel intensity profiles (mean ± SD, n = 8 ribbons) across the center of ribbons before (black), immediately after (red), and 250 s after (blue) bleaching. Ribbons diameters were normalized to the same width; the peaks of pixel intensities were normalized. (**f**) Average recovery of the bleached hemisphere (from c) compared to particle simulations with different diffusion coefficients (colored traces). Each simulation trace is an average of 20 runs. See also Supplementary Fig. S2, Supplementary Movie S1.

A major challenge in imaging ribbon synapses arises from the ribbon’s size being comparable to or smaller than the diffraction-limited resolution of light microscopy (~250 nm). This limitation makes it challenging to resolve the dynamics within ribbons in live-imaging preparations. Here, to bypass this limitation, we exploit the larger size of ribbons (~1 µm) in neuromast hair cells of the lateral line system of larval (4–6 day old) transgenic *ribeye b*-EGFP zebrafish^25^. At these ages, larvae have a functional lateral line system and hair cells exhibit robust synaptic output^25, 26^. Evidence suggests that most hair cells are capable of mechanotransduction^27^, and the majority of ribbons appose postsynaptic elements (Supplementary Fig. S1). Each neuromast cluster (Fig. 1a,b) comprises 10–14 hair cells, with each hair cell containing ~3 spherical ribbons (full width at half maximum [FWHM] of *ribeye b*-EGFP ribbons in our data set: 798 ± 194 nm, mean ± SD, n = 39 ribbons). The results are presented in two main parts: 1) estimating the overall time course of Ribeye turnover and exchange at single ribbons and between the hair cell’s entire ribbon population (Fig. 1); 2) measuring the mobility of Ribeye within single ribbons as a gauge of structural dynamism (Fig. 2).

### Turnover and exchange of Ribeye at ribbons

Overall, the *in vivo* local turnover and exchange of synaptic proteins in the nervous system remains poorly understood. For our purposes, we define “local turnover” and “exchange” as the replacement of a protein both within (“local turnover”) and between (“exchange”) synapses. At conventional synapses, postsynaptic proteins such as transmitter receptors and scaffolding molecules exhibit protein half-lives of t_1/2_ ≈ 2–15 hours and apparent local turnover half-times (assessed via FRAP) of t_1/2_ ≈ 1 minute (for review, see ref. 28). Does Ribeye, the main structural protein of the presynaptic ribbon, exhibit similar time courses? Replacement of Ribeye at the synapse could be due to 1) unstable synaptic ribbons (or chunks of ribbon) that detach and reattach in such a way that Ribeye is swapped out, or 2) ongoing exchange of Ribeye at stably-rooted ribbons to gradually replace the local stock of protein. To test these possibilities, we first determined the spatial stability of ribbons, and then specifically measured the turnover and exchange of Ribeye. For these experiments, we monitored Ribeye b-EGFP fluorescence for many ribbons across an entire neuromast (~10–14 hair cells; Fig. 1a,b). Data sets comprised confocal *z*-stacks encompassing the volume of the neuromast (3-D), acquired once every 60 s for 6000 s, and were processed into maximum intensity projections (2-D) for each time point. We then registered the images, tracked ribbon position, and measured Ribeye b-EGFP fluorescence during the time course.

To understand the spatial stability of ribbons, we tracked the X-Y locations of ribbons throughout the neuromast during the 6000 s imaging period to determine the movements of whole ribbons. For this analysis, we distinguished between the momentary spatial “stability” of ribbons (i.e. how much they wiggle in place) versus the overall drift of ribbons (i.e. directed movement over time). Data for 12 ribbons (same experiment as Fig. 1b) are shown in Fig. 1c,d (see also Supplementary Fig. S1). As a measure of momentary stability, instantaneous displacements (Fig. 1c) were calculated for each ribbon as the change in X-Y location between time points during the 6000 s time course. As exemplified by three colored examples (Fig. 1c), we observed heterogeneous stability patterns between ribbons within the same neuromast and observation period. For example, ribbons showed periods of greater movement bracketed by periods of relatively little movement, sometimes hundreds of seconds in duration (Fig. 1c). However, overall most instantaneous displacements were small and weakly correlated across ribbons (Average Pearson’s R: 0.13; Supplementary Fig. S1). Do these periods of higher ribbon instability (as measured by instantaneous displacement) result in the drift of ribbon locations over time? Over the course of an experiment (6000 s), the average displacement of ribbons from their original X-Y starting location was 0.59 ± 0.33 µm (mean ± SD, n = 45 ribbons; Fig. 1d, Supplementary Fig. S1), a rate of drift considerably less (roughly 10 times) than observed for dissociated bipolar cell ribbons^6^. For perspective, momentary displacements between successive time points equal to one third to one half of this final value (0.59 µm) were relatively common in our datasets. Together, these data suggest that ribbons *in vivo* can experience bouts of spatial instability (i.e. wiggling in place) while remaining largely stationary with little drift within a hair cell across long timescales.

After determining the stability of hair cell ribbons *in vivo*, we examined Ribeye turnover and exchange at ribbons. For these experiments, we completely bleached a few selected Ribeye b-EGFP ribbons (no more than one bleached ribbon per hair cell; Fig. 1b) at the beginning of the long-term imaging experiments and monitored the recovery of fluorescence. By the end of the 6000 s imaging period, Ribeye b-EGFP at bleached ribbons recovered 25 ± 2.3% (n = 9, mean ± SD; Fig. 1e). Extrapolating an exponential recovery from the averaged data (to 100% of pre-bleach level) gives an estimated recovery time constant τ = 6.9 h. Extrapolating exponential recoveries from individual ribbon data, and removing the assumption of a 100% recovery, resulted in an average estimated time constant of 1.3 ± 1.1 h (range: 0.4–4.0 h) and a maximum recovery to 33 ± 16% (range: 21–70%) of pre-bleach levels (mean ± SD). These hours-long timescales of recovery are broadly consistent with observed postsynaptic protein half-lives^28^, leaving the source of fluorescence recovery ambiguous: either newly synthesized fluorescent Ribeye or an exchange of unbleached Ribeye between neighboring synapses might underlie the recovery. In principle, these two potential sources of recovery can be distinguished by monitoring the unbleached ribbons sharing the cell with a bleached ribbon. If Ribeye exchange occurs between ribbons, then the fluorescence of the unbleached ribbons should decrease as it receives bleached Ribeye from the bleached ribbon. On the contrary, if fluorescence recovery arises exclusively from newly synthesized fluorescent Ribeye, then the fluorescence of unbleached ribbons should not decrease. Therefore, to determine whether the recovery of bleached ribbons was at least partially due to exchange of Ribeye between ribbons of the same cell, we monitored the fluorescence of unbleached ribbons, both within the same cell of the bleached ribbon (teal) and in neighboring cells where no ribbons were bleached (purple, Fig. 1b,e). By 6000 s after bleaching, a statistically significant difference accumulated between these two unbleached ribbon populations (p < 0.0001 at time 5991 s, student’s t test), with the unbleached ribbons that shared the same cell as a bleached ribbon losing more fluorescence. Plotting the difference between these populations ([unbleached, other cell]-[unbleached, same cell], dashed black in Fig. 1e,f) shows a gradual increase over time, suggesting that a hair cell’s ribbons can exchange Ribeye between synapses. By the end of the 6000 s imaging period, this difference between unbleached ribbon populations reached ~20%. Further quantification of the relative contributions of Ribeye exchange from neighboring ribbons within the same cell proved difficult, and the size of the bleached ribbon did not seem to strongly influence recovery characteristics (Supplementary Fig. S1). We did observe small, more mobile Ribeye puncta (perhaps analogous to the “precursor spheres” observed in electron microscopy^16–18, 29^), which on rare occasions seemed to merge with larger, presumably synaptically-located ribbons. However, none of the observed recovery from bleaching in our experiments was due to fusion of these small puncta. Therefore, over hours-long time periods, ribbons can apparently exchange Ribeye via a relatively diffuse reservoir of Ribeye in the cytoplasm. Additionally, and in contrast to the relative transience of postsynaptic proteins (t_1/2_ ≈ 1 minute)^28^, Ribeye appears to have a relatively low turnover at individual ribbon synapses.

### Mobility of Ribeye within ribbons

At cultured hippocampal (non-ribbon) synapses, postsynaptic scaffold molecules appear to be highly plastic in time, with protein distributions within a postsynaptic density morphing on a timescale of minutes^30^. Similarly, at ribbon synapses, electron microscopic snapshots in time suggest that ribbons are structurally dynamic during specific contexts (e.g. development, hibernation, diurnal cycles), when dramatic morphological changes take place. However, these electron microscopic observations have a temporal resolution that is relatively poor across sub-minute time periods, and provide no details concerning the ongoing, steady-state internal dynamics of the ribbon’s ultrastructure – only changes in overall ribbon morphology. As a result, real-time *in vivo* structural properties of ribbons are almost completely unknown. The ribbon is considered to be a presynaptic scaffold^31^, but does the same dynamic mobility seen within the postsynaptic scaffold also occur presynaptically for the ribbon structure? More specifically, are the ribbon’s constituent proteins (the majority of which are the protein Ribeye) rigidly bound to each other, or are they more loosely associated with each other? To probe the stability or mobility of Ribeye b-EGFP within the ribbon, we bleached one hemisphere of an individual ribbon and monitored the fluorescence intensity of the bleached and non-bleached sides in continuously-acquired, time-lapse confocal images focused at the ribbon’s center (red, black, and gray boxes in Fig. 2a). After bleaching one hemisphere of a ribbon, the average total fluorescence lost across the whole ribbon (red box in Fig. 2a,b) was 58 ± 13% (mean ± SD, n = 19 ribbons). We used two approaches to analyze FRAP data: 1) an approach that minimized noise by averaging many pixels in order to measure FRAP time courses (Fig. 2a–c); and 2) an approach with better spatial resolution at the expense of fine temporal precision (Fig. 2d,e).

Using the first approach, we found that the bleached side recovered <1 minute after bleaching (τ = 41.6 s exponential fit, n = 21 ribbons, Fig. 2c), and that the non-bleached side dimmed (with stable overall fluorescence). Considering an average fluorescence loss of 58% from the whole ribbon during bleaching, full recovery of the bleached side would only reach 42% of the pre-bleaching intensity as the bleached and unbleached sides equilibrate with the remaining fluorescent Ribeye. By 250 s after bleaching, however, the bleached side only recovered to 27%, suggesting that only 64% of Ribeye is mobile (See Supplementary Movie S1). To determine whether fluorescence recovery was dependent on synaptic activity, we repeated these experiments after blocking presynaptic L-type calcium channels with isradipine. The recovery time course was nearly identical (τ = 43.4 s, n = 21 ribbons; Supplementary Fig. S2), suggesting observed Ribeye mobility may be independent of synaptic activity.

Using the second approach, we tracked the change in intensity through time for each pixel location in a dataset during the early phase of recovery (*i* immediately before bleaching, *ii* immediately after bleaching, and *iii* changes that occurred during the first 50 frames, or ~17 s, after bleaching; Fig. 2d). During recovery, in the cytoplasm surrounding the ribbon, pixels on average did not become brighter or darker (gray values in Fig. 2d *iii*). However, we observed that pixels on the bleached side became brighter (lighter values) and pixels on the unbleached side became dimmer (darker values), indicating that fluorescence recovery was due to unbleached Ribeye moving across to the bleached half of the ribbon (Fig. 2d *iii*). If ribbons contained entirely mobile Ribeye, then the fluorescence profile across a ribbon would reassume the same shape after recovering from bleaching as it had before bleaching. Conversely, ribbons containing entirely immobile Ribeye would maintain the same fluorescence profile as observed immediately after bleaching. In our experiments, the overall shape of the fluorescence profile after 250 s more closely resembled the original profile before bleaching than after bleaching (Fig. 2e), consistent with our above calculation that ~64% of the Ribeye is mobile.

Mobility of Ribeye within the ribbon could be random or directed. To see if random diffusion could account for our observations, we created simulations of Ribeye diffusion within a ribbon (see Supplementary Methods and Supplementary Fig. S2). In these simulations, we created spherical ribbons with the same average diameter as observed in our FRAP datasets and filled them with particles initially confined to only one hemisphere. At time 0 s, the barrier between hemispheres was removed, allowing particles to diffuse anywhere in the sphere, while counts were made in boxes corresponding to regions measured in FRAP experiments (Fig. 2a). The effective diffusion coefficient (D) for particles that best fit our FRAP data was 0.0016 µm^2^/s (τ = 40 s, Fig. 2f). In contrast, the predicted diffusion coefficient of free (i.e. non-ribbon) Ribeye b-EGFP is 49.5 µm^2^/s [calculated^32^ for 93.2 kDa Ribeye b^33^ plus the 27 kDa EGFP tag]. Collectively, our data suggests that, although turnover of Ribeye at a single ribbon is an hours-long process (Fig. 1), the mobility of Ribeye proteins within the ribbon is high – the majority (64%) of Ribeye appears to be mobile, and capable of moving across the ribbon structure in less than a minute. As a result of the ~500× slower rate of recovery of completely bleached ribbons (Fig. 1) relative to within-ribbon recovery (Fig. 2), it is unlikely that our within-ribbon recovery data is confounded with Ribeye exchange with the cytoplasm. Lastly, our simulations suggest that this Ribeye mobility within the ribbon could be mediated by a random diffusion process, although due to Ribeye-Ribeye interactions^31^ and steric effects in the crowded internal environment of the ribbon: 1) a more complex mechanism may be at play, and 2) such diffusion within the ribbon would be dramatically (~31,000×) hindered compared to free Ribeye.

## Discussion

In this study, we examined the structural dynamics of ribbons (via monitoring dynamics of the main protein Ribeye). While most imaging studies of live ribbon synapse activity have utilized dissociated retinal cells^6, 34–43^, relatively little is known about hair cell ribbons^35, 44^ or how ribbons behave *in vivo*. By utilizing *ribeye b*-GFP transgenic zebrafish, we were able to investigate hair cell ribbons *in vivo*, for long durations, and without disrupting the structural integrity, osmolarity, or mechanical forces surrounding the cells (which may influence calcium influx and synaptic activity^45^). The most striking observation in our data concerns the large difference in Ribeye mobility within the ribbon compared to Ribeye turnover at the synaptic level.

First, our findings suggest a considerably slower overall turnover rate for synaptic Ribeye (~6–7 hours; Fig. 1) than observed elsewhere at postsynaptic densities, on par with the protein half-life of other synaptic proteins^28^. As the dominant constitutive protein of ribbons, this long residence of Ribeye protein at the synapse may serve to stabilize the synapse - perhaps an important characteristic for a sensory synapse that must constantly and reliably encode presynaptic membrane potential. Our data showing relatively small spatial displacements over relatively long periods (~0.5 µm over the course of 100 minutes - less than the diameter of the ribbon, Fig. 1) further supports this idea of synaptic stability. However, in stark contrast to the apparent longer-term stability, our data also revealed shorter-term instability (e.g. bouts of wiggling in place, Fig. 1) and a high mobility of the ribbon’s underlying structure (Fig. 2). This was unexpected, as the dense, regular packing of proteins in plate-like retinal ribbons^46^, combined with the ribbon’s proposed roles as a presynaptic scaffold, evokes a sense of physical rigidity. On the contrary, the Ribeye mobility we observed within the ribbon is curiously supportive of a “fluid mosaic” model of the ribbon surface. One could speculate that such a fluid ribbon surface might allow vesicle-bound tethers to diffuse laterally within a sea of Ribeye, providing an alternate hypothesis of vesicle delivery to the presynaptic membrane to “crowd surfing” along location-fixed tethers^47^.

There are a number of possible explanations for the differences between our observations and previous work, including: fundamental structural differences between plate-like and spherical ribbons, or between hair cell and retinal ribbons; differences arising from different animal models; and differences between *in vivo* and *in vitro* conditions. As imaging technology advances, future studies with higher resolution, higher sensitivity, faster imaging speeds, and more advanced fluorescent probes will hopefully reveal ever greater insight and detail towards the dynamic nature of ribbon synapses and their associated vesicles.

## Methods

### Fish care and sample preparation

Zebrafish (*Danio rerio*) work was performed under animal study protocol #1362–13 approved by the Animal Care and Use Committee at the NIH. Strains were maintained in TAB5 or Tubingen wild-type background. Zebrafish larvae were raised in E3 embryo media (5 mM NaCl, 0.17 KCl mM, 0.33 mM CaCl_2_ and 0.33 mM MgSO_4_) at 30 °C. Larvae were examined at 4–6 days post fertilization (dpf). To image ribbon synapses, the previously described transgenic line *Tg*(*-6.5myosin6b:Ribeye b-GFP*)*vo67* was used^25^. Despite having enlarged ribbons compared to wildtype siblings, transgenic fish have no observable auditory or vestibular defects, and therefore represent a viable model to study ribbon dynamics. For our analyses only basally localized ribbons were examined, rather than small ectopic cytosolic Ribeye b-EGFP aggregates. For microscopy, larvae were anaesthetized with 0.01% ethyl 3-aminobenzoate methanesulfonate (MS-222, Sigma-Aldrich). Larvae were then oriented in glass-bottomed dishes for imaging in 1% low melting point agarose and immersed in E3 embryo media containing 0.01% MS-222. To block synaptic function, 10 μM isradipine (Sigma-Aldrich) and 0.1% DMSO was added to the low melting point agarose and the E3 solution.

### FRAP acquisition and analysis

FRAP experiments were performed on a Zeiss LSM 780 inverted confocal microscope with a 63×/1.4 NA oil objective lens. For FRAP, a rectangular region (edge ≈ 1–2 µm) was drawn to encompass half of the ribbon or the entire ribbon. Bleaching was performed with a 405 nm laser, while pre- and post-photobleaching images were acquired using a 488 laser line for Ribeye b-GFP. A previous study used bright illumination of a fluorescein-based Ribeye-binding peptide to inactivate Ribeye protein via fluorophore-assisted light inactivation (FALI)^9^. Because 1) our bleaching times were much shorter (~1 s vs. 15–60 s for FALI), and 2) GFP is considerably less effective at generating free radicals than fluorescein^48^, we assume that inactivation of our Ribeye b-EGFP did not have a strong influence on Ribeye function in our FRAP experiments. All time series data sets (i.e. FRAP datasets in Figs 1 and 2) were acquired at 88 nm pixel size. For most datasets, a single image focal plane was set through the center of the ribbon; drift in the z-dimension in and out of the focal plane was typically not a problem for short datasets (e.g. <5 minutes). However, for a subset (n = 5) of experiments (Fig. 2), we continuously acquired *z*-stacks of the whole ribbon throughout the experiment (1 stack every ~4 s) to determine drift in the *z*-dimension and to compare maximum intensity projections of these stacks to single-frame datasets. We concluded that *z*-dimension drift was minimal and that single-frame datasets would be sufficient. For long datasets (6000 s, Fig. 1), *z*-stacks were acquired every minute that encompassed the whole or most of the neuromast. During acquisition, the neuromast would slowly drift, requiring brief pauses (~1 minute) to re-center the acquisition. This re-centering was required 2–4 times per dataset.

#### Acquisition frame rates

Short datasets (e.g. <5 minutes, Fig. 2) were acquired at 4.17 ± 0.48 (ave ± SD) frames per second, with a subset at ~0.25 frames per second (as stacks, see above), and another subset (n = 10) at ~23 frames per second. Datasets across these acquisition rates showed similar recovery curves and were combined.

#### Measurement regions

For Ribeye b-EGFP FRAP measurements, measurement regions were as depicted in Fig. 2a. To measure the same relative positions on ribbons of varying size, each ribbon was divided into fifths, and the measurement region was one fifth wide by three fifths tall.

Data was processed in ImageJ and IgorPro (WaveMetrics), and diffusion simulations were run in Smoldyn 2.36 (www.smoldyn.org)^49^.

## Electronic supplementary material

Supplementary Information

Supplementary Movie S1
