# Supplementary material for: In Vivo Ribbon Mobility and Turnover of Ribeye at Zebrafish Hair Cell Synapses
Source: Sci Rep. 2017 Aug 7;7:7467. doi: 10.1038/s41598-017-07940-z (PMC5547071; doi:10.1038/s41598-017-07940-z)
Supplement: Supplementary file 1 — Supplementary Information [file 41598_2017_7940_MOESM1_ESM.pdf]

Supplementary Information

Title: *In Vivo* Ribbon Mobility and Turnover of Ribeye at Zebrafish Hair Cell Synapses

Authors: Cole W. Graydon, Uri Manor, and Katie S. Kindt

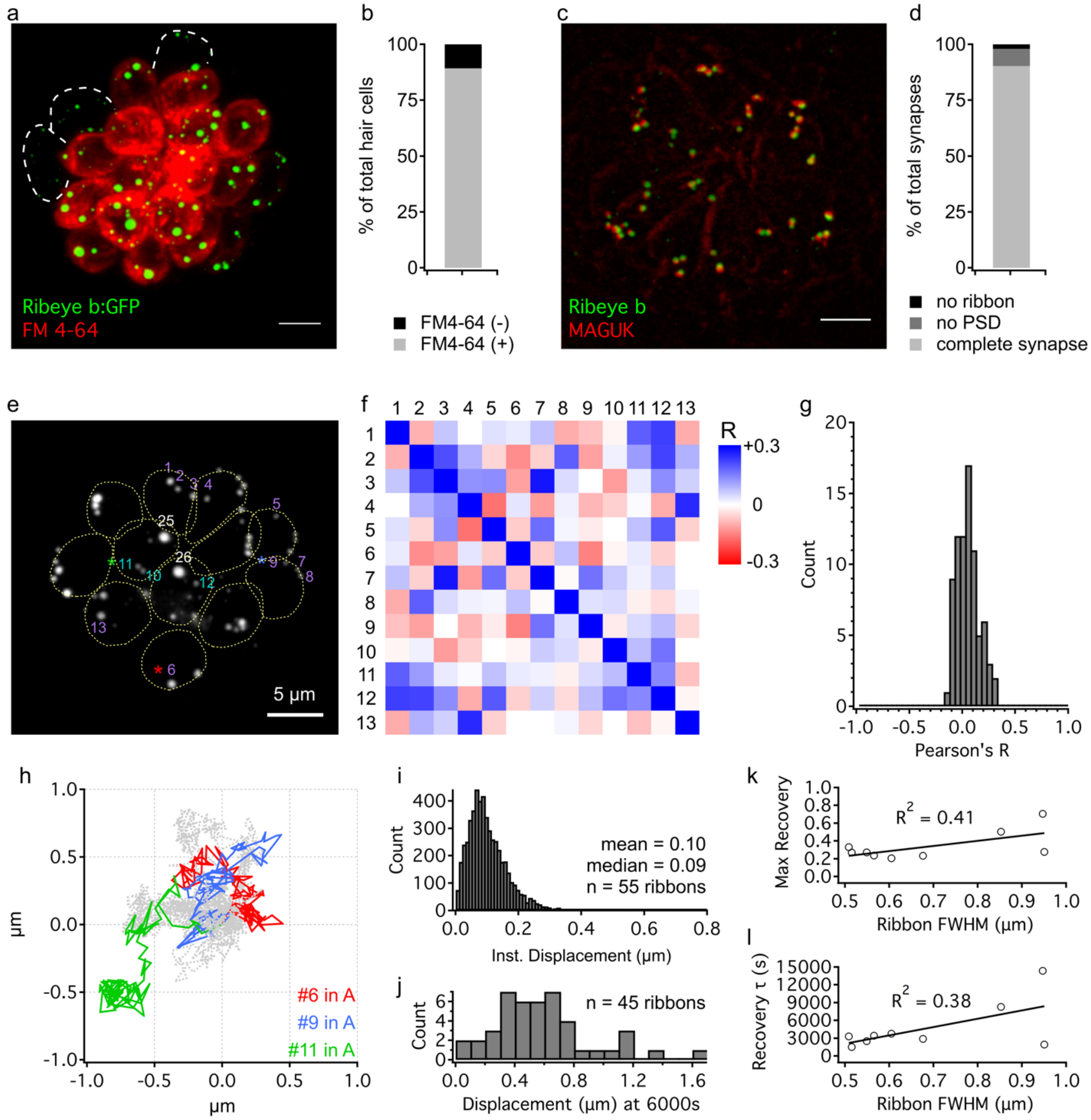

Supplementary Figure S1 (Related to Fig. 1) - Tracking hair cell ribbon positions over long time scales.
a) Example neuromast showing cytoplasmic labeling of *ribeye b-GFP* hair cells following bath application of FM4-64 (red) to indicate cells competent for mechanotransduction. Unlabeled hair cells are outlined with dashed lines.
b) Quantification of the number of hair cells that label with FM4-64. n= 176 hair cells from 4 fish.
c) Example neuromast with antibody labeling of Ribeye b (green) and the postsynaptic marker MAGUK (red).
d) Quantification of the number of hair cell ribbons with postsynaptic (MAGUK) elements. n= 388 complete or partial synapses.
e) Example neuromast with ribbons numbered (same as Fig. 1b).
f) Plot of Pearson's correlation coefficients between ribbons numbered in e, color coded with positive correlations in blue and negative correlations in red.
g) Distribution of all correlation coefficients plotted in e.
h) x-y displacements through time for numbered ribbons in e and f (each ribbon started at 0,0 at time 0s).

i) Distribution of instantaneous displacement values calculated for each ribbon at each time point.
j) Distribution of final displacements after 6000s for all ribbons tracked in four separate experiments (n=45 ribbons).
k) Plot of the maximum recovery (from exponential fit of FRAP data) against the diameter (FWHM) of the bleached ribbon (n=9). Linear fit shown with coefficient of determination (R^2).
l) Plot of the recovery time constant τ (from exponential fit of FRAP data) against the diameter (FWHM) of the bleached ribbon (n=9). Linear fit shown with coefficient of determination (R^2).

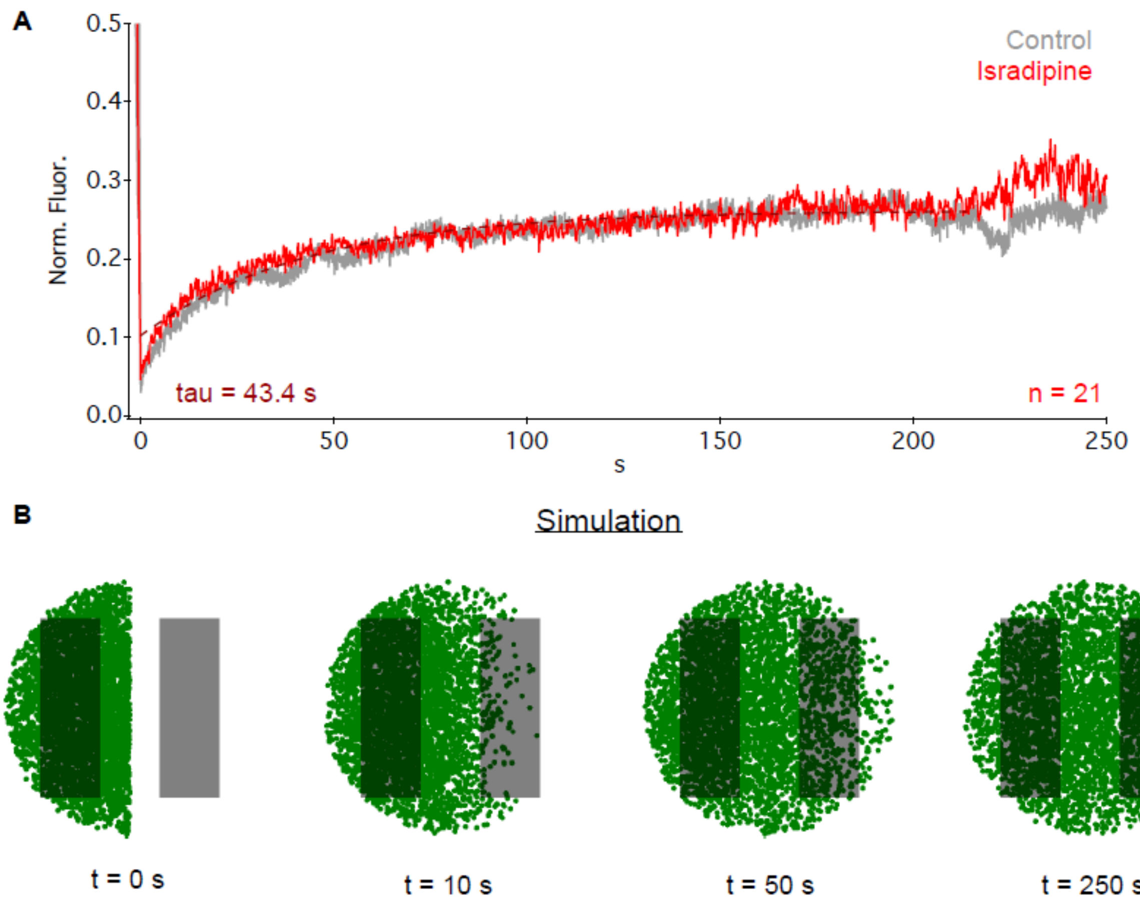

Supplementary Figure S2 (Related to Fig. 2) - Effect of isradipine on Ribeye fluorescence recovery and diffusion simulation example.

A) Comparison of normalized average fluorescence recovery following bleaching for control condition (same as Fig. 2c, gray, n=21) and 10 µM isradipine condition (red, n=21).

B) Four simulation snapshots at different time points, showing the position of diffusing particles (green) relative to measurement boxes (gray rectangles).

Supplementary Movie S1 (Related to Fig. 2) - Examples of half-bleached ribbon fluorescence recovery
Three examples are shown side-by-side, with a pixel size of 88nm. Bleaching of half of the ribbon occurs after 10 frames. The approximate frame rate for acquisition was ~4 frames/s. At the end of the movie, average images (Before, After, and Recovery) are shown. Before averages the 10 frames immediately preceding the bleach. After averages the 10 frames immediately following the bleach. Recovery averages 100 frames starting approximately 200s after the bleach.

Supplementary Methods

Image processing and analysis of short data sets (Fig. 2)
During image acquisition, care was taken to maximize the dynamic range of the image and to avoid saturated pixels. After acquisition, image sets were cropped, scaled, aligned, and shifted. For each of these steps, the original image pixel values for each time point were preserved (i.e. there was no spatial or temporal filtering).

Cropping: This was used to make dataset file sizes smaller and isolate the ribbon of interest in cases where more than one ribbon was contained in the field of view. This was useful for more computationally intense routines during alignment step.
Scaling: Images were scaled up 5x, meaning that each pixel (88nm) was turned into a 5x5 group of subpixels (17.6nm each) with the same value as the original pixel. This scaling allowed much finer alignments between successive images in dataset. For example, if the calculated centers of the ribbon in two successive images were off in one dimension by 40 nm, the alignment process would shift the second image by 2 subpixels (or 35.2 nm), resulting in an error of 4.8 nm. In the original image sets, no alignment would be possible because 40 nm is less than half the size of a pixel.
Alignment: Over the time course of acquiring of a short data set (30-300s), ribbons would typically have subtle x-y drifts within the image. To correct for these drifts, we wrote custom image processing routines in Igor Pro. For alignment, each Ribeye b-EGFP fluorescence image in a data set was fit with a 2D Gaussian function. Next, starting with the first image after bleaching as the base image, subsequent images were shifted so that the centers of the Gaussian fits were in register.
Shifting: The use of 2D Gaussian functions for alignment was excellent for correcting x-y drift, but because the first image after bleaching was used as the base image for alignment, the difference between the center of the ribbon prior to bleaching and after bleaching was maintained throughout recovery. As a result, the center of the fully recovered ribbon was offset from the original, pre-bleaching ribbon center. To correct for this, we shifted the ribbon image as a function of the fluorescence recovery so that when the ribbon was fully recovered, the before- and after-bleach centers of the ribbon were in register.

Image processing and analysis of long data sets (Fig. 1)
Datasets consisted of image stacks (3D) for each time point, and were processed into a time series of 2D maximum intensity projections. Image registration of these time series was done using ImageJ's StackReg algorithm using "rigid body" alignment so that images were not spatially distorted. StackReg minimizes the mean-square difference of intensities between successive images, using image n as an anchor for image $n+1$, and the initial image of the stack alignment as a global anchor. Ribbon x-y positions and intensities (Fig. 1c-f; Supplementary Fig. S1) were tracked through time using the SpotTracker 2D ImageJ plugin[1] (http://bigwww.epfl.ch/sage/soft/spottracker/).

Normalization and time constant calculation
Normalized fluorescence intensity of FRAP recovery (Fig. 1e,f; Fig. 2b,c,e,f; Supplementary Fig. S2) was calculated by setting the average fluorescence intensity for the 3-10 frames before bleaching to 1.0, and setting the lowest intensity achieved within ~3 frames following bleaching to 0. In Figure 2e, both the height (i.e. max fluorescence intensity) and width of ribbon profiles were normalized so that the central value of a Gaussian fit to the profile was 1.0 (height), and profile widths were rescaled so that ribbons of different diameters could be grouped. For less noise and better fits, recovery τs (Fig. 1e,f; Fig. 2c; Supplementary Fig. S2) were calculated based on single-exponential fits of recovery averages.

Diffusion simulations (Fig. 2f; Supplementary Fig. S2)
All diffusion simulations were run in Smoldyn 2.36 software[2] (www.smoldyn.org). Simulation parameters were selected to recreate the typical ribbon geometries and measurement regions of our experiments (see Fig. 2a). To this end, ribbons were simulated as 1150 nm diameter spheres, corresponding to the average experimental FWHM of

Ribeye b-EGFP fluorescence profiles (798 nm; n = 38 ribbons from imaging data sets) after accounting for the airy disk. Simulation time steps were 10ms, yielding average particles step lengths of 2.8-6.3 nm per time step for the diffusion coefficients (0.0004 to 0.002 $\mu m^2 s^{-1}$ in steps of 0.0004) examined. Each simulation had three structures: the ribbon sphere that contained all diffusing molecules, a dividing plane that passed through the center of the ribbon sphere and served as a diffusion barrier between the two halves, and a transparent counting box that did not hinder diffusing particles. The counting box had the same relative size and position as the region used to make experimental measurements in imaging data sets. At the start of a simulation, 4000 diffusing particles were created in one half of the ribbon sphere (created by the dividing plane) and allowed to equilibrate to uniform density for 150 simulated seconds. After equilibration, the dividing plane was removed and particles were free to diffuse anywhere within the ribbon sphere (denoted t=0s). For the next 400 seconds of simulated time, the number of particles was counted in the counting box at each time step. Twenty simulations were run for each diffusion coefficient, and the results were averaged and scaled for comparison to experimental data in Figure 2f. Particles in our Smoldyn simulations had no volume and could not react with each other, and as a result were not influenced by steric or concentration effects. Therefore, the diffusion coefficients in simulations should be considered as "effective diffusion coefficients" that encapsulates these effects. Simulation data was analyzed in Igor Pro software.

Immunohistochemistry to label neuromast ribbon synapses (Supplementary Fig. S1)
Zebrafish larvae at day 5 were fixed with 4% paraformaldehyde in phosphate-buffered saline (PBS) for 4 hours at 4°C. Larvae were then permeabilized with ice cold acetone for 4 min, and blocked with PBS containing 2% goat serum, 1% bovine serum albumin (BSA) overnight at 4°C. Primary antibodies were diluted in PBS containing 1% BSA, and larvae were incubated 4 hours at room temperature. A custom made primary antibody for Ribeye b (IgG2a, 1:10,000), and a commercially available antibody for Membrane-associated Guanylate Kinases (MAGUK, IgG1, 1:500, NeuroMab AB_10698179) have been described and used previously[3]. After removal of primary antibodies, larvae were incubated in diluted secondary antibodies coupled to Alexa 488 or Alexa 647 (Life Technologies) for 3 hours. After removal of secondary antibodies, larvae were mounted in ProLong Gold Antifade reagent and imaged on a Zeiss LSM 780 inverted confocal microscope (see main text) using 488 and 647 nm lasers.

FM4-64 dye labeling to assay mechanotransduction (Supplementary Fig. S1)
To visualize hair cells competent for mechanotransduction, *ribeye b-GFP* larvae at day 5 were immersed in a 3 µM solution of FM4-64, (Life Technologies) in E3 for 30 seconds, and immediately washed 4 times in E3. Larvae were mounted in 1% low melt agarose and imaged on Zeiss LSM 780 inverted confocal microscope (see main text) using 488 and 594 nm lasers.

Supplementary References

References

1 Sage, D., Neumann, F. R., Hediger, F., Gasser, S. M. & Unser, M. Automatic tracking of individual fluorescence particles: application to the study of chromosome dynamics. *IEEE Trans Image Process* **14**, 1372-1383 (2005).

2 Andrews, S. S., Addy, N. J., Brent, R. & Arkin, A. P. Detailed simulations of cell biology with Smoldyn 2.1. *PLoS Comput Biol* **6**, e1000705, doi:10.1371/journal.pcbi.1000705 (2010).

3 Sheets, L., Trapani, J. G., Mo, W., Obholzer, N. & Nicolson, T. Ribeye is required for presynaptic Ca(V)1.3a channel localization and afferent innervation of sensory hair cells. *Development* **138**, 1309-1319, doi:10.1242/dev.059451 (2011).
